# Breast Desmoid Tumours: A Review of the Literature

**DOI:** 10.1155/2024/5803290

**Published:** 2024-07-12

**Authors:** Mike Wu, Thomas Michael Hughes, Senarath Edirimanne, Nicholas Ngui

**Affiliations:** ^1^ James Cook University, Townsville, QLD 4810, Australia; ^2^ Department of Vascular Surgery Townsville University Hospital, Townsville, QLD 4814, Australia; ^3^ Australian National University School of Medicine and Psychology, Canberra, ACT 2600, Australia; ^4^ Sydney Adventist Hospital Breast and Soft Tissue Oncology Services, Wahroonga, NSW 2076, Australia; ^5^ Department of Surgery Nepean Hospital, Kingswood, NSW 2747, Australia; ^6^ Sydney University, Camperdown, NSW 2006, Australia; ^7^ Department of Surgery Blacktown-Mount Druitt Hospital, Mount Druitt, NSW 2770, Australia

## Abstract

Breast desmoid tumour is a rare type of benign breast disease that presents like malignancy. Current guidelines are based on limited evidence derived from case reports and small case series and recommend resection with microscopically-negative margin (R0). There is a high risk of recurrence despite negative surgical margins. A review of the published cases of breast desmoid since 2000 was conducted using Medline and Embase to descriptively analyse the clinical presentation, diagnosis, treatment, and outcomes of this rare disease. After screening, we identified 46 patients from 39 articles. Most cases did not have risk factors, but 17/46 (37%) had prior procedures on the ipsilateral breast. Mammography was able to detect 65% of the cases, ultrasound detected 74%, and both CT and MRI detected all cases when used. Preoperative diagnosis was best performed using core needle biopsy showing typical histology and positive beta-catenin staining. 42/46 cases underwent definitive surgical management, with 8 cases of recurrence. Recurrence occurred within 3 years of the initial surgery. Median time of recurrence was 8 months, and the median follow-up of the recurrence-free patients was 12 months. There were no predictive factors identified for recurrence. There were 7 cases treated with a nonsurgical modality, with 3 showing at least a partial response.

## 1. Introduction

Desmoid tumour (DT), also known as “aggressive fibromatosis,” was first coined in 1838 by Muller, after the Greek word “desmos,” meaning “tendon” or “band.” It describes a disease that histopathologically appears as monoclonal fibroblastic proliferations arising from musculo-aponeurotic structures [1]. Clinically, it presents as a hard palpable mass or with pain when it arises deep and compresses nearby structures.

Desmoid tumours are rare tumours that have been reported as occurring in most parts of the body. Most commonly, they occur in the hip and shoulder girdles, the abdominal wall, and within the abdominal cavity [2]. Intra-abdominal DT is most frequently found in the mesentery of the small intestine. These intra-abdominal tumours are often associated with adenomatous polyposis coli (APC) gene mutation and familial adenomatous polyposis (FAP). The majority (80%) of extraabdominal DT have a mutation in the beta-catenin gene leading to nuclear accumulation of the beta-catenin protein. The APC and beta-catenin mutations are thought to be mutually exclusive [3]. The absence of beta-catenin should raise suspicion of APC mutation-related DT even in extraabdominal sites.

DT may develop as a firm, well-defined nodule. In other cases, the mass may be poorly defined. In either case, DT is histologically characterised by microscopic extensions with infiltrating tentacles extending well beyond the mass [4]. The disease can be multifocal and multicentric but it does not metastasise [5]. In most cases, growth is progressive but in up to 15% of the cases, spontaneous regression will occur. Due to the rarity of DT, our knowledge of the natural history and outcomes of management are based on retrospective case series. A curious finding is that local recurrence does not necessarily occur following a microscopically incomplete (R1) resection [6].

DT can occur in the breast, affecting females more often than males, and sometimes presenting bilaterally. It is very rare and makes up an extremely small proportion of all breast tumours (0.2%) [7]. The first cases reported were associated with Lynch syndrome. Currently, the main risk factor is prior surgery in the same anatomical field [8], making up 44% of the presentations in one institution-based retrospective study. It is unlikely that estrogen or progesterone exposure would play any role in the genesis of breast DT as it does not express any of these sex hormone receptors [9, 10], an important difference between breast desmoids and other desmoid tumours in which estrogen receptors are expressed in up to 33% of the cases [11].

Due to the rarity of breast DT, it is an under-recognised differential diagnosis in the preoperative workup, with many patients proceeding to surgery for “discordance” in the triple assessment for breast cancer. The recommended treatment of breast DT is wide local excision to achieve negative margins [12]. The reported rate of recurrence after surgery is 18–27%, with positive margins seeming to increase the risk [13], but recurrence can also occur after R0 resection [8]. Surgical resection has the potential to cause significant morbidity particularly for deep-seated DT that invades the chest wall. In such cases, nonsurgical options have been used [14, 15].

This study aims to investigate the reported treatment and outcomes for breast DT over the last 20 years, with particular assessment of the risk factors for recurrence. Breast DT can develop in deeper tissue planes and border the chest wall. When this occurs, it is difficult to distinguish whether the origin was from skeletal muscle or breast parenchymal tissue. This study also aims to investigate whether these would comprise two different pathologies, an idea which has been considered by the literature but not specifically examined.

## 2. Materials and Methods

**Figure 1:**
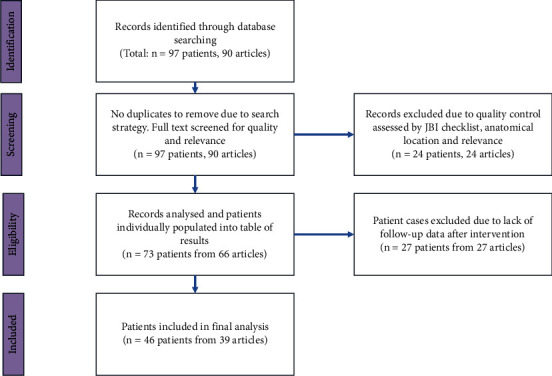
Flow diagram for the retrieval and selection of articles from the literature search.

A literature review was conducted using Medline and Embase on July 3, 2022. A combination of MeSH headings and keywords were combined to maximise the articles captured using a search strategy that is included in Appendix 1. The inclusion criteria were all reports of fibromatosis confined to the breast and axilla region, published after 2002. Results were limited to case reports with full English text available. The initial search was performed by a single researcher and returned 90 results, with details on 97 patients. The full text of all 90 case reports was obtained and screened by one researcher. Articles were also excluded if there were several aspects missing from the case report. Cases which were of dubious quality were assessed using the Joanna Briggs Institute Checklist for Case Reports (Appendix 2). Any article scoring less than 6 out of the 8 criteria were excluded from the analysis. From the 90 results, 24 articles on 24 patients were excluded. 3 did not meet the JBI standards, 7 did not meet anatomical requirements (outside of the breast or inside the area of the breast but purely chest wall lesions), 12 were about a different disease, and 2 were irrelevant to the topic. This resulted in 66 articles on 73 patients. 27 of these articles were missing follow-up information on the outcome of intervention, so the final dataset includes 39 articles on 46 patients (see Figure 1). Statistical analysis and design of figures was performed on Microsoft Excel 365.

### 2.1. Data Collection

Demographic information was collected from each patient case including age, gender, and risk factors for developing fibromatosis. Familial syndromes, previous trauma, surgery, or augmentation were captured. Gravidity, parity, menopause, and use of hormone replacement therapy information were also collected.

Data were collected related to presentation and diagnosis. This included relationship of disease to nearby structures (whether there was clinical or radiological evidence that the disease was tethered to the chest wall), features of locally advanced disease, the imaging modalities used for detection and the results of these tests, whether a noninvasive biopsy was performed before surgery and the type of sampling employed, whether fibromatosis was diagnosed before surgery, and whether diagnostic immunohistochemistry was performed on the biopsy specimen or the surgical specimen.

Information was collected on interventions and outcomes. This included the type of intervention, the extent of surgical resection (excisional biopsy vs. wide local excision vs. mastectomy vs. radical resection), neoadjuvant or adjuvant therapy, requirement for reoperation, and the resection margins after definitive treatment. The outcomes assessed in this study were recurrence-free survival, time until recurrence after surgical treatment, and disease reduction/progression after nonsurgical treatment. All included case reports were required to report the follow-up duration.

## 3. Results

### 3.1. Demographics

There were 3 males and 43 females. The median age was 36.5, and the range was 21–75. If the cases were grouped by age into decades, the proportion of cases in the age 30–40 category is the highest, at 17%.

### 3.2. Risk Factors for Fibromatosis

**Figure 2:**
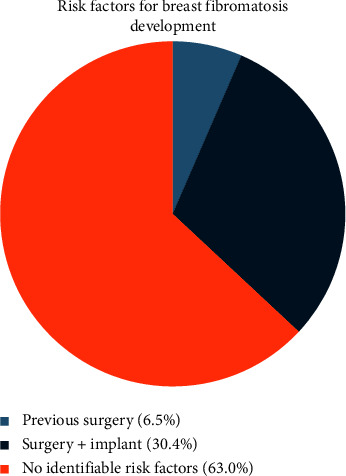
The proportion of breast desmoids cases with known risk factors.

While most cases arose de novo, previous instrumentation with or without breast implant was the only identifiable risk factor in this study population. Among the 46 cases, 17 (37%) had previous procedures on the ipsilateral breast. Of these 17, 14 had breast implants in situ at the time of diagnosis (Figure 2).

For the 17 cases that occurred after surgery and/or implant placement, the median age was 33. For the other 29 cases that arose de novo, the median age was 37. There was a trend for cases related to prior surgery to be in younger patients, but this was not statistically significant (*p*=0.18). For the 17 patients who had a record of their previous surgery or implant insertion, the median time until diagnosis of fibromatosis was 24 months (range: 12–48 months).

### 3.3. Chest Wall vs. Intraparenchymal Disease

15 cases were purely intraparenchymal, 24 cases were documented to involve the chest wall, and in 7 cases, the anatomical location of the DT was not specified. Of the 15 intraparenchymal cases, 14 were not associated with any risk factors. Of the 24 chest wall cases, 15 were associated with prior surgery. There were a total of 17 cases associated with prior surgery ± implant.

### 3.4. Locally Advanced Disease

9/46 (20%) cases presented with features of locally advanced disease, defined as presence of skin tethering, skin dimpling, skin infiltration, or nipple retraction.

### 3.5. Detectability on Imaging

Mammography was used in 30 (65%) of the 46 patients. In 8 (27%) instances, the disease was not detected, and half of these were associated with implants. Ultrasound was used in 34 (74%) of the 46 patients. In one instance, the disease was not detected, and this was associated with an implant. If used, CT and MRI usually occurred after an initial attempt with mammography and ultrasound. CT was used in 9/46 cases; 4 cases were because initial imaging was negative, 2 cases were to obtain more information, and in 3 cases, it was used directly as the first imaging modality. MRI was used in 17/46 cases; 4 cases were used because initial imaging was negative, 11 cases were to obtain more information, in 2 cases, it was used directly as the first modality. CT and MRI detected all DT when used.

### 3.6. Tissue Sampling and IHC and Their Effect on the Rate of Preoperative Diagnosis

**Figure 3:**
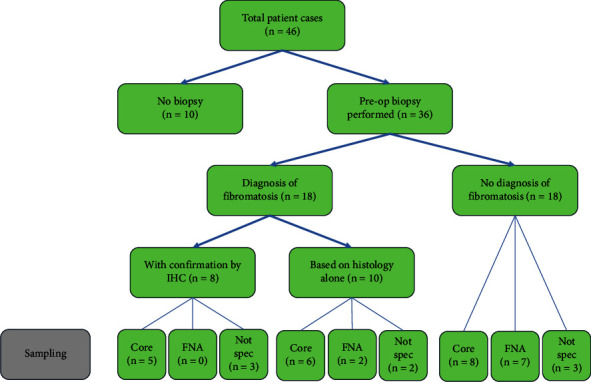
Diagnosis of breast DT.

Figure 3 summarises the pathway to diagnosis for the 46 cases. 10 cases proceeded directly to surgery for excision biopsy with no preoperative percutaneous biopsy. 36 cases had an attempted percutaneous biopsy, from which 18 diagnoses of breast DT were made. 9 fine needle biopsies were performed with the diagnosis of DT being made in 2 (22%) cases. 19 core biopsies were performed with the diagnosis of DT being made by histology alone in 6 cases and with histology and immunohistochemistry in 5 cases. In 8 cases, the nature of the percutaneous biopsy was not specified, and 5 cases were diagnosed with breast DT. For the 28 cases for which there was not a definite diagnosis of DT from percutaneous biopsy, the diagnosis was established on the excised specimen with immunohistochemistry in 19 cases and by histology alone in 9 cases. IHC was diagnostic of DT in all 8 percutaneous biopsy specimens on which it was performed. IHC staining for beta-catenin in association with histological appearance is considered diagnostic for breast DT. DT is further characterised by positive staining for vimentin, actin, and desmin and negative staining for CD34.

### 3.7. Treatment

DT was resected in 42 of the 46 cases. In 40 of these, there was no neoadjuvant or adjuvant treatment. One surgical patient had initially been treated with tamoxifen and underwent resection due to tumour progression. One surgical patient had postoperative radiotherapy for positive surgical margins. In the other 4 cases, nonsurgical methods were the only therapeutic modality.

### 3.8. Types of Resections

In the 42 patients who underwent therapeutic DT resection, there were 48 procedures performed. The final procedure performed was local excision (*n* = 28), mastectomy (*n* = 5), and radical resections that involved the chest wall (*n* = 9). There were 6 patients who underwent two surgical procedures; 1 case underwent initial incisional biopsy and proceeded to have a second definitive procedure, 3 cases underwent initial diagnostic excision and went on to require a wider excision, and 2 cases underwent wide resection which required further excision. Thus, of the 28 local excisions, 5 (18%) required re-excision.

### 3.9. Outcomes

#### 3.9.1. Recurrence, Progression, and Disease-Free Survival

**Figure 4:**
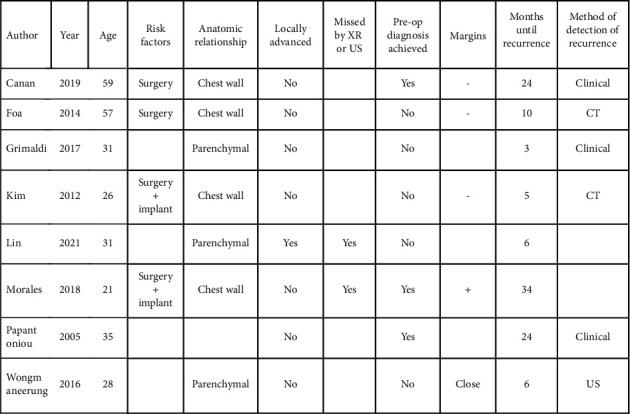
The 8 cases of recurrence after resection and their risk factors.

Measurable outcomes of the treatment modalities were reported as recurrence (return of disease after resection), progression (increase in volume of disease after medical treatment), or disease-free survival (length of time of follow-up during which the patient was negative for the disease). Of the 42 cases that underwent definitive surgical management, there were 8 recurrences (Figure 4). All the recurrences occurred within 3 years of the surgical procedure. For the patients that experienced recurrence, the median time until recurrence was 8 months. For the patients that were recurrence-free, the median follow-up time was 12 months. Recurrence can be detected clinically or radiologically.

**Figure 5:**
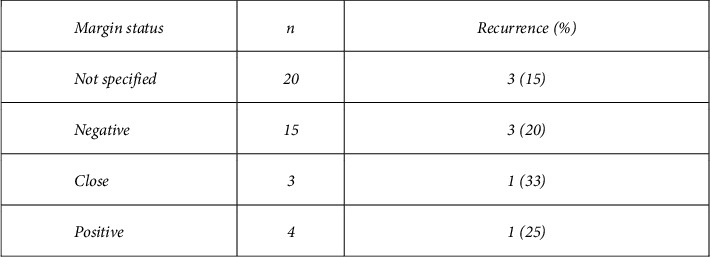
Outcome by the surgical margin status for the 42 patients who underwent definitive surgical treatment.

The margin status was reported in 22 of the 42 cases that underwent surgery for definitive management (Figure 5). They were reported as either positive, negative, or “close.” 3 of the 15 cases with surgically negative margins resulted in recurrence (median follow-up: 24 months). 2 of the 7 cases with surgically positive or close margins had recurrence (median follow-up: 18 months). The remaining 3 cases of recurrence occurred in the 20 patients whose margin status at surgery was not reported (median follow-up: 6 months).

There is a lack of predictive factors for recurrence, besides previous surgery and implant procedure. Factors that may be associated with recurrence, e.g., anatomic relationship, presence of locally advanced features, being radiologically occult, preoperative index of suspicion, and residual disease; all did not have any association with recurrence.

#### 3.9.2. Reoperation

There were 5 cases that underwent reoperation due to close or positive margins after initial planned complete excision. The diagnosis was unknown prior to the initial surgical procedure in all these cases. In 4 cases, preoperative biopsy was by FNA and was nondiagnostic in all. The type of biopsy in the remaining case was not specified but it did show a spindle cell lesion of unknown malignant potential. Of these 5 cases, 1 resulted in recurrence.

#### 3.9.3. Nonsurgical Modalities

**Figure 6:**
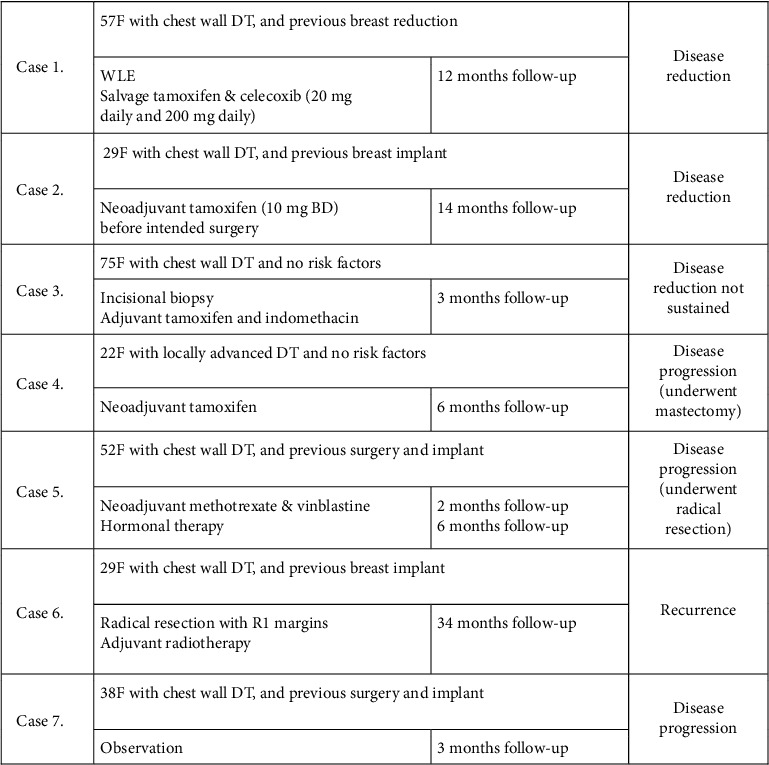
7 cases of nonsurgical therapies and their outcomes.

There were 7 patient cases in which a nonsurgical modality such as drug or radiotherapy was employed as part of the treatment, with a follow-up to assess effect (Figure 6). Of these 7 cases, 6 of them were associated with DT involving the chest wall. In 3 of these cases, there was at least a partial response.

## 4. Discussion

### 4.1. Causation

In this study, there was no significant difference in age between the groups of fibromatosis cases that arose de novo compared with cases that followed surgery and/or implant insertion. The overall median age of 36.5 is consistent with the age of onset of DT at other anatomical sites [2, 3]. There was a strong temporal relationship between the surgical procedure and presentation with fibromatosis, with a median lag time of 24 months (range: 12–48 months). This suggests there could be a causative relationship, with surgery and/or an implant stimulating an abnormal fibroblast reaction. Breast DT is a rare potential side effect of breast surgery that all patients who are considering breast augmentation or reconstruction should be aware of.

### 4.2. Clinical Presentation

Breast DT presents as a painless firm lump, which can be mobile or tethered to skin or chest wall. There is no nipple discharge, but there may be local features such as nipple retraction or skin dimpling as was the case in 20% of the cases presented. It is not associated with axillary lymphadenopathy. At presentation, the priority of the clinician is to exclude malignancy due to the often suspicious clinical and radiological features. Although very rare, the diagnosis of DT should be considered as an alternate diagnosis to breast carcinoma.

### 4.3. Diagnostic Imaging

Breast DT can appear as a stellate lesion on mammography and may contain microcalcifications, an appearance usually associated with primary breast carcinoma [16]. On ultrasound, it appears as an irregular hypoechoic mass with indistinct margins and posterior acoustic shadowing [17]. In this study of 46 patients, the sensitivity of mammography and ultrasound were 73% and 97%, respectively. All cases of DT were visible on cross-sectional imaging with CT and MRI. CT and MRI were used to assess for local extension and to identify surgical planes. The appearance of DT on MRI imaging is variable and does not distinguish DT from malignant disease [18].

### 4.4. Tissue Diagnosis

Tissue diagnosis following initial imaging is essential to establish the diagnosis and differentiate the lesion from primary breast carcinoma and other pathology. Fine needle biopsy is not adequate to establish a definitive diagnosis in the majority of the cases. Percutaneous image-guided core biopsy will be diagnostic in a significant portion of patients.

There was a lack of information in many of the cases reported but when core biopsy was performed, the diagnosis was established either by routine microscopic examination or with the additional aid of immunohistochemistry in 8 (44%) cases. In cases where core biopsy fails to establish a definite diagnosis, excision biopsy should be considered. The histological appearance of DT is a lesion with sheets of uniform, spindle-shaped fibroblasts forming sweeping or interlacing fascicles entrapping ducts and lobules with an infiltrative edge. The degree of cellularity varies [16]. There are trapped lymphocytes interspersed. In areas of previous surgery, it can be hard to distinguish from scar tissue. Immunohistochemistry is the most accurate means of establishing the diagnosis. Breast DT is positive for beta-catenin, smooth muscle actin, and vimentin and negative for cytokeratin, estrogen, progesterone, and androgen receptors [10, 19]. It is also negative for CD34 [20]. It must be interpreted by an experienced pathologist to rule out the differential diagnoses [21].

In this study, 10 patients did not undergo biopsy and proceeded directly to excision. 6 of these patients had breast implants, and while the reasons were not stated, it may have been that biopsies were not performed due to inability to access the lesion.

In the 5 cases that required two or more operations, the second operation was performed due to close or positive margins, and for all of them, the diagnosis of DT had not been made before the first operation. If a preoperative diagnosis had been made in these cases, multiple procedures may have been avoided. It is likely that the treating surgeon would have taken wider margins which would likely have included the microscopic extensions. This highlights the importance of obtaining a preoperative diagnosis with core biopsy and use of immunohistochemistry in all suspected cases to allow planning of appropriate surgical resection and providing patients with the most accurate advice possible.

The optimal management of breast DT has not been established. There are studies which demonstrate a proportion of patients with extraabdominal DT being observed without surgery with stable symptoms and occasionally spontaneous regression [22]. In this review, there was only one case where the initial management was observation. The DT progressed at 3 months of follow-up, and the case report did not specify what the next step in management would be. Current guidelines recommend the surgical resection of fibromatosis using wide margins [23]. In the absence of any other evidence, this seems very reasonable providing such treatment would not create excessive morbidity.

This accumulated case series provides no evidence to support the use of adjuvant radiotherapy or primary treatment with medical therapies. The recommendations for nonsurgical treatment modalities, including radiotherapy, NSAIDs, tamoxifen, and chemotherapy, are based on literature about extramammary DT [14]. Breast DT appears to possibly have a different phenotype and as a result may not respond to nonsurgical modalities as would be expected with extramammary DT. Features suggestive of a different phenotype to other DT include lower recurrence rates [24], negative staining for estrogen, progesterone, and androgen receptors, and lower response rates to NSAIDs, chemotherapy, or hormonal therapy [9]. Tamoxifen has been shown to be potentially effective through a mechanism proposed by Ishizuka [25]. This was not validated in the few cases in which this was used in this accumulated series. Radiotherapy was only used in one case, as adjuvant therapy when there were positive surgical margins. This was unsuccessful and disease recurrence eventuated at 34 months of the follow-up. Due to the lack of evidence to support their efficacy for breast DT, the use of nonsurgical modalities should be reserved for second-line use, when surgical resection would be an excessively morbid procedure due to disease location or patient factors or as adjuvant treatment for R1 resections. In this study, there were only a few cases of nonsurgical modalities or modalities employing surgery and adjuvant therapies, and their numbers are too small to draw any conclusions on effect.

Due to the large number of patients who underwent surgical management, this study provides insight into surgical approaches and outcomes. Godwin [12] proposed that radical surgery achieving clear margins is the surest way of eradicating the disease and proposed a protocol for its management. This can be extremely successful, as demonstrated by a recent study published by Lorenzen, showing no recurrence in a series of 15 patients managed surgically with negative margins at a single institution over the last 10 years [26]. However, it is acknowledged that recurrence is unpredictable and can occur despite negative resection margins. This has been supported by more recent studies [27]. In the current study, the rate of recurrence was 8/43 (18.6%). Negative margins were not protective of recurrence, as 3 of the 8 recurrences had negative margins. The literature encourages achieving negative margins, as the risk of recurrence is higher when surgical margins are positive [8] compared to when margins are negative. The findings of the current study support this conclusion. The reporting of margin status was poor with only 22 of the 42 definitive surgical cases stating the status. In these 22 cases, 2 of the 7 cases with “close” or positive margins experienced recurrence, compared to only 3 out of the 15 cases with reported negative margins, suggesting higher risk of recurrence if margins are positive. However, these rates must be interpreted cautiously due to risk of reporting bias. There are no other reliable predictors of recurrence. Histological features have not been shown to be predictive for recurrence [4]. Other factors that could influence recurrence such as age, previous surgery or implant, relationship to local anatomy, features of locally advanced disease, poor detectability by X-ray or ultrasound, whether diagnosis was made from the biopsy, and margin status after resection were examined in this study, but none of these factors showed a strong correlation with recurrence (Figure 4).

There is no consensus on the appropriate width of surgical margins, and some aim to achieve a margin of 2-3 cm [28]. None of the reviewed studies indicated what their criteria were for reporting surgical margins as “negative” or “close.” Therefore, it is difficult to compare these results and apply them externally. Among the group of 8 recurrences, 4 were excisions, 3 were WLE, and 1 was a radical resection. The recurrence rate for each category of resection was 4/13 for excision, 3/15 for WLE, 0/5 for mastectomy, and 1/9 for radical resection. This suggests that wider excision margins do reduce the risk of recurrence, with the most favourable being mastectomy and radical resection. Consideration must be made to morbidity and patient preference. If a patient prefers not to undergo a repeat procedure after the initial one has positive margins, then observation is quite reasonable as there is a significant chance that their disease will not progress. Satoko et al. [29] refer to a case of spontaneous regression despite positive margins. In this study, spontaneous regression of residual disease occurred in 2 of the 3 cases with residual disease after surgical resection which was followed up with observation. There was no progression of disease at 12 and 36 months of follow-up. There was one case of “close” surgical margins which was followed up by observation and demonstrated disease progression at 6 months follow-up.

It has been suggested that breast DT can originate from either the muscular aponeurosis, making it similar to extramammary DT, or from the parenchyma itself [30, 31]. It is relevant because extramammary fibromatosis originating from aponeurosis seems to be more responsive to hormonal therapy, and this may apply even though mammary fibromatosis does not express estrogen receptors. In this study, 3 of the cases demonstrated disease reduction after nonsurgical therapy. This suggests that medical therapy (tamoxifen or NSAIDs) should be considered for breast desmoids that have contact with the chest wall. For all the cases in this study that were purely intraparenchymal disease, medical therapy was not attempted, and to our knowledge, there are no reports of its success. In this series, recurrence rates were similar between aponeurotic-based and parenchymal-based DT with 3/15 intraparenchymal cases and 4/24 chest wall cases having recurrence. It has been suggested that intraparenchymal recurrence is higher than aponeurotic recurrence [9].

## 5. Conclusion

Breast DT is a rare tumour that should be considered a differential diagnosis for lesions suspicious both clinically and radiologically of breast carcinoma. This is particularly the case for patients with breast implants. Patients having breast implants inserted should be warned of the possibility of the development of this rare tumour. Preoperative tissue diagnosis should be established with core biopsy examination with the use of immunohistochemistry. Following diagnosis, imaging with CT or MRI can be very helpful for treatment planning. The most effective treatment is complete surgical excision with clear margins. Involved margins do not necessitate reexcision; however, patients do need to be monitored for at least 3 years as this is the time in which recurrence is likely to occur. Primary medical therapy does not appear to be helpful. Adjuvant radiotherapy following surgery is not recommended.

This is a review of literature from the last 20 years in order to provide a descriptive analysis of the clinical presentation, diagnosis, treatment, and outcomes of this rare disease.

## Data Availability

The data used to support the findings of this study are available from the corresponding author upon reasonable request.

## Abbreviations

ER: Estrogen receptor
FNA: Fine needle aspirate
IHC: Immunohistochemistry
JBI: Joanna Briggs Institute
NSAID: Nonsteroidal anti-inflammatory drug
PR: Progesterone receptor
WLE: Wide local excision.

## Supplementary Materials

Supplementary Materials Appendix 1 contains the search strategy used on the Medline and Embase databases. Appendix 2 contains the Joanna Briggs Institute checklist for case reports, which was used to test the quality of case reports before inclusion into our dataset for analysis. Appendix 3 contains a table of the journal articles that contributed patients towards this review, the data collected from these articles, and their citations.
