# Supplementary material for: Breast Desmoid Tumours: A Review of the Literature
Source: Breast J. 2024 Jul 12;2024:5803290. doi: 10.1155/2024/5803290 (PMC11259505; doi:10.1155/2024/5803290)
Supplement: Supplementary Materials — Appendix 1 contains the search strategy used on the Medline and Embase databases. Appendix 2 contains the Joanna Briggs Institute checklist for case reports, which was used to test the quality of case reports before inclusion into our dataset for analysis. Appendix 3 contains a table of the journal articles that contributed patients towards this review, the data collected from these articles, and their citations. [file 5803290.f1.zip › Appendix 1 - Search strategy.docx]

Appendix 1: Search strategy

Medline - 64 results

1. (Breast or mammary).tw.

2. exp Breast/

3. 1 or 2

4. Fibromatosis, Aggressive/

5. (desmoid adj3 fibromatosis).tw.

6. 4 or 5

7. 3 and 6

8. (breast adj4 fibromatosis).tw.

9. 7 or 8

10. limit 9 to english language

11. limit 10 to yr="2002 -Current”

12. limit 11 to case reports

Embase - 35 results

1. (breast or mammary).tw.

2. exp breast/

3. 1 or 2

4. exp fibromatosis/

5. exp desmoid tumor/

6. (desmoid$ adj3 fibromatosis).tw.

7. 4 or 5 or 6

8. 3 and 7

9. limit 8 to (english language and yr="2002 -Current")

10. exp case report/

11. 9 and 10

12. limit 11 to exclude medline journals

9 irretrievable articles: Al-Saleh, Arora, Catalano, Ferbeyre, Jamshed, Kouriefs, Long, Mazzocchi, Sachdev
