# Supplementary material for: Breast Desmoid Tumours: A Review of the Literature
Source: Breast J. 2024 Jul 12;2024:5803290. doi: 10.1155/2024/5803290 (PMC11259505; doi:10.1155/2024/5803290)
Supplement: Supplementary Materials — Appendix 1 contains the search strategy used on the Medline and Embase databases. Appendix 2 contains the Joanna Briggs Institute checklist for case reports, which was used to test the quality of case reports before inclusion into our dataset for analysis. Appendix 3 contains a table of the journal articles that contributed patients towards this review, the data collected from these articles, and their citations. [file 5803290.f1.zip › Appendix 2 - JBI checklist for case reports.docx]

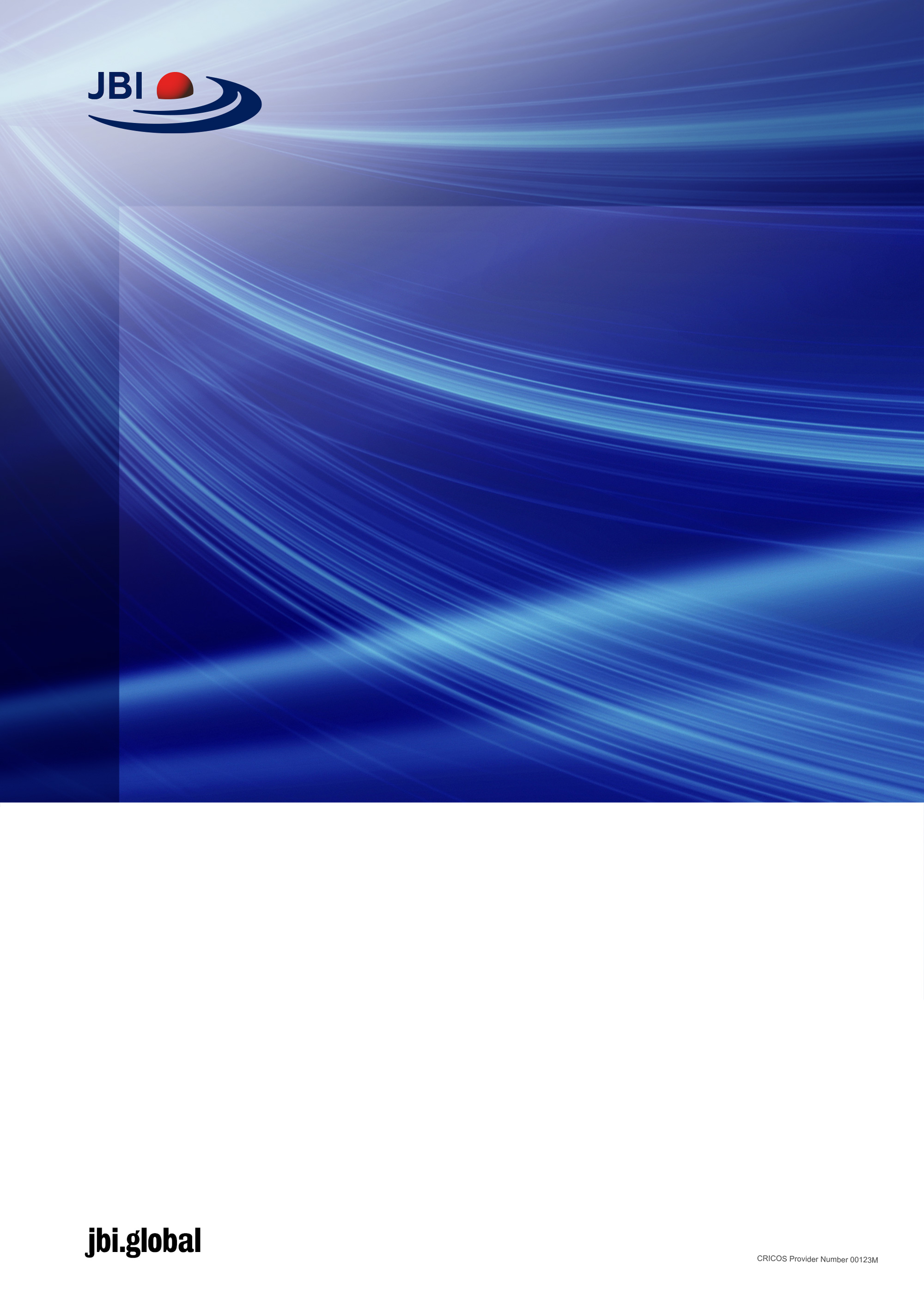


checklist for case reports

Critical Appraisal tools for use in JBI Systematic Reviews

Introduction

JBI is an international research organisation based in the Faculty of Health and Medical Sciences at the University of Adelaide, South Australia. JBI develops and delivers unique evidence-based information, software, education and training designed to improve healthcare practice and health outcomes. With over 70 Collaborating Entities, servicing over 90 countries, JBI is a recognised global leader in evidence-based healthcare.

## JBI Systematic Reviews

The core of evidence synthesis is the systematic review of literature of a particular intervention, condition or issue. The systematic review is essentially an analysis of the available literature (that is, evidence) and a judgment of the effectiveness or otherwise of a practice, involving a series of complex steps. JBI takes a particular view on what counts as evidence and the methods utilised to synthesise those different types of evidence. In line with this broader view of evidence, JBI has developed theories, methodologies and rigorous processes for the critical appraisal and synthesis of these diverse forms of evidence in order to aid in clinical decision-making in healthcare. There now exists JBI guidance for conducting reviews of effectiveness research, qualitative research, prevalence/incidence, etiology/risk, economic evaluations, text/opinion, diagnostic test accuracy, mixed-methods, umbrella reviews and scoping reviews. Further information regarding JBI systematic reviews can be found in the [JBI Evidence Synthesis Manual](https://jbi-global-wiki.refined.site/space/MANUAL).

## JBI Critical Appraisal Tools

All systematic reviews incorporate a process of critique or appraisal of the research evidence. The purpose of this appraisal is to assess the methodological quality of a study and to determine the extent to which a study has addressed the possibility of bias in its design, conduct and analysis. All papers selected for inclusion in the systematic review (that is – those that meet the inclusion criteria described in the protocol) need to be subjected to rigorous appraisal by two critical appraisers. The results of this appraisal can then be used to inform synthesis and interpretation of the results of the study. JBI Critical appraisal tools have been developed by the JBI and collaborators and approved by the JBI Scientific Committee following extensive peer review. Although designed for use in systematic reviews, JBI critical appraisal tools can also be used when creating Critically Appraised Topics (CAT), in journal clubs and as an educational tool.

JBI Critical Appraisal Checklist for case reports

Reviewer ______________________________________ Date_______________________________

Author_______________________________________ Year_________ Record Number_________

|  | Yes | No | Unclear | Not applicable |
| --- | --- | --- | --- | --- |
| 1. Were patient’s demographic characteristics clearly described? | □ | □ | □ | □ |
| 1. Was the patient’s history clearly described and presented as a timeline? | □ | □ | □ | □ |
| 1. Was the current clinical condition of the patient on presentation clearly described? | □ | □ | □ | □ |
| 1. Were diagnostic tests or assessment methods and the results clearly described? | □ | □ | □ | □ |
| 1. Was the intervention(s) or treatment procedure(s) clearly described? | □ | □ | □ | □ |
| 1. Was the post-intervention clinical condition clearly described? | □ | □ | □ | □ |
| 1. Were adverse events (harms) or unanticipated events identified and described? | □ | □ | □ | □ |
| 1. Does the case report provide takeaway lessons? | □ | □ | □ | □ |

Overall appraisal: Include □ Exclude □ Seek further info □

Comments (Including reason for exclusion)

________________________________________________________________________________________________________________________________________________________________________________________________________________________________________________________________________________________________

Explanation of case reports critical appraisal

*How to cite: Moola S, Munn Z, Tufanaru C, Aromataris E, Sears K, Sfetcu R, Currie M, Qureshi R, Mattis P, Lisy K, Mu P-F. Chapter 7: Systematic reviews of etiology and risk. In: Aromataris E, Munn Z (Editors)*. JBI Manual for Evidence Synthesis. JBI, 2020. Available from <https://synthesismanual.jbi.global>

**Case Reports Critical Appraisal Tool**

Answers: Yes, No, Unclear or Not/Applicable

## Were patient’s demographic characteristics clearly described?

Does the case report clearly describe patient's age, sex, race, medical history, diagnosis, prognosis, previous treatments, past and current diagnostic test results, and medications? The setting and context may also be described.

## Was the patient’s history clearly described and presented as a timeline?

A good case report will clearly describe the history of the patient, their medical, family and psychosocial history including relevant genetic information, as well as relevant past interventions and their outcomes. (CARE Checklist 2013)

## Was the current clinical condition of the patient on presentation clearly described?

The current clinical condition of the patient should be described in detail including the uniqueness of the condition/disease, symptoms, frequency and severity. The case report should also be able to present whether differential diagnoses was considered.

## Were diagnostic tests or methods and the results clearly described?

A reader of the case report should be provided sufficient information to understand how the patient was assessed. It is important that all appropriate tests are ordered to confirm a diagnosis and therefore the case report should provide a clear description of various diagnostic tests used (whether a gold standard or alternative diagnostic tests). Photographs or illustrations of diagnostic procedures, radiographs, or treatment procedures are usually presented when appropriate to convey a clear message to readers.

## Was the intervention(s) or treatment procedure(s) clearly described?

It is important to clearly describe treatment or intervention procedures as other clinicians will be reading the paper and therefore may enable clear understanding of the treatment protocol. The report should describe the treatment/intervention protocol in detail; for e.g. in pharmacological management of dental anxiety - the type of drug, route of administration, drug dosage and frequency, and any side effects.

## Was the post-intervention clinical condition clearly described?

A good case report should clearly describe the clinical condition post-intervention in terms of the presence or lack thereof symptoms. The outcomes of management/treatment when presented as images or figures would help in conveying the information to the reader/clinician.

## Were adverse events (harms) or unanticipated events identified and described?

With any treatment/intervention/drug, there are bound to be some adverse events and in some cases, they may be severe. It is important that adverse events are clearly documented and described, particularly when a new or unique condition is being treated or when a new drug or treatment is used. In addition, unanticipated events, if any that may yield new or useful information should be identified and clearly described.

## Does the case report provide takeaway lessons?

Case reports should summarize key lessons learned from a case in terms of the background of the condition/disease and clinical practice guidance for clinicians when presented with similar cases.

References:

Gagnier JJ, Kienle G, Altman DG, Moher D, Sox H, Riley D, CARE Group. The CARE Guidelines: Consensus‐Based Clinical Case Reporting Guideline Development. Headache: The Journal of Head and Face Pain, 2013;53(10):1541-1547.
