# Supplementary material for: Breast Desmoid Tumours: A Review of the Literature
Source: Breast J. 2024 Jul 12;2024:5803290. doi: 10.1155/2024/5803290 (PMC11259505; doi:10.1155/2024/5803290)
Supplement: Supplementary Materials — Appendix 1 contains the search strategy used on the Medline and Embase databases. Appendix 2 contains the Joanna Briggs Institute checklist for case reports, which was used to test the quality of case reports before inclusion into our dataset for analysis. Appendix 3 contains a table of the journal articles that contributed patients towards this review, the data collected from these articles, and their citations. [file 5803290.f1.zip › Appendix 3 - List of included articles.docx]

| Author | Year | n | Age | Sex | Risk factors | Anatomic relationship | Imaging used | IHC diagnosis pre-op? | Curative procedure | Non-surgical therapies | Margins | Follow-up (months) | Recurrence |
| --- | --- | --- | --- | --- | --- | --- | --- | --- | --- | --- | --- | --- | --- |
| Al-Khyatt (Al-Khyatt et al., 2010) | 2010 | 1 | 22 | F |  |  | US | N | Mastectomy | Y |  | 36 | N |
| Al-Yusuf (Al-Yusuf et al., 2005) | 2005 | 1 | 46 | F |  | Chest wall | XR | N | WLE |  | Negative | 32 | N |
| Benej (Benej et al., 2017) | 2017 | 2 | 36 | F |  | Chest wall | XR, US, CT | Y | WLE |  | Negative | 10 | N |
|  |  |  | 43 | F |  | Chest wall | XR, US, CT | Y | WLE |  |  | 8 | N |
| Bouab (Bouab et al., 2022) | 2022 | 1 | 51 | F | Prior surgery | Parenchymal | XR, US | Y | WLE |  | Negative | 10 | N |
| Brown (Brown et al., 2012) | 2012 | 1 | 29 | F |  | Parenchymal | XR, US, MRI | N | Mastectomy |  | Negative | 9 | N |
| Canan (Canan & Wang, 2019) | 2019 | 1 | 59 | F | Prior surgery | Chest wall | XR, US | Y | WLE |  | Negative | 24 | Y |
| Chummun (Chummun et al., 2010) | 2010 | 1 | 22 | F | Implant | Chest wall | US, MRI | N | Excision |  |  | 5 | N |
| Collins (Collins et al., 2017) | 2017 | 1 | 44 | F | Implant | Chest wall | CT | Y | WLE |  | Negative | 36 | N |
| Foa (Foa et al., 2014) | 2014 | 1 | 57 | F | Prior surgery | Chest wall | CT | N | WLE |  | Negative | 10 | Y |
| Gergele (Gergele et al., 2012) | 2012 | 1 | 38 | F | Implant | Chest wall | US, MRI | Y | N/A (x`observation) | |  | 3 | N/A |
| Greenberg (Greenberg et al., 2002) | 2002 | 1 | 53 | F |  | Parenchymal | XR, US | N | WLE |  |  | 24 | N |
| Grimaldi (Grimaldi et al., 2018) | 2018 | 1 | 31 | F |  | Parenchymal | US, MRI | N | WLE |  |  | 3 | Y |
| Hammood (Hammood et al., 2021) | 2021 | 1 | 59 | F |  | Parenchymal | XR, US | N | WLE |  |  | 6 | N |
| Hammoudeh (Hammoudeh & Darian, 2012) | 2012 | 1 | 38 | F | Implant |  | MRI | N | Excision |  |  | 12 | N |
| Henderson (Henderson et al., 2010) | 2010 | 1 | 27 | F | Implant | Chest wall |  | N | Excision |  |  | 24 | N |
| Horevoets (Horevoets et al., 2013) | 2013 | 1 | 67 | F |  | Chest wall | XR, US, MRI | Y | WLE |  | Negative | 3 | N |
| Jung (Jung et al., 2010) | 2010 | 1 | 46 | F |  | Parenchymal | XR, US | Y | WLE |  |  | 12 | N |
| Karahan (Karahan et al., 2017) | 2017 | 1 | 32 | F |  | Chest wall | US | N | WLE |  | Negative | 54 | N |
| Khanfir (Khanfir et al., 2003) | 2003 | 1 | 52 | F | Implant | Chest wall | CT, MRI | N | Excision | Y | Positive | 8 | N/A |
| Kim (Kim et al., 2012) | 2012 | 1 | 26 | F | Implant | Chest wall | US, MRI | N | Excision |  | Negative | 5 | Y |
| Lee (Lee et al., 2015) | 2015 | 1 | 32 | F |  | Parenchymal | US | N | WLE |  | Negative | 17 | N |
| Lin (Lin et al., 2021) | 2021 | 1 | 31 | F |  | Parenchymal | XR (neg), US | N | WLE |  |  | 6 | Y |
| Matherne (Matherne et al., 2004) | 2004 | 2 | 50 | F |  | Parenchymal | XR, US | N | Excision |  |  | 72 | N |
|  |  |  | 31 | F |  | Chest wall | XR, US | N | Excision |  |  | 12 | N |
| Meshikhes (Meshikhes et al., 2005) | 2005 | 1 | 37 | M |  | Parenchymal | XR, US | N | Mastectomy |  |  | 12 | N |
| Morales (Morales et al., 2018) | 2018 | 4 | 21 | F | Implant | Chest wall | XR (neg), CT, MRI | Y | WLE | Y | Positive | 34 | Y |
|  |  |  | 31 | F | Implant | Chest wall | XR (neg), CT, MRI | Y | WLE |  | Negative | 36 | N |
|  |  |  | 33 | F | Implant | Chest wall | XR (neg), CT, MRI | Y | WLE |  | Negative | 24 | N |
|  |  |  | 42 | F | Implant | Chest wall | XR (neg), CT | Y | WLE |  | Negative | 18 | N |
| Munhoz (Munhoz et al., 2017) | 2017 | 1 | 30 | F | Implant | Chest wall | US (neg), MRI | N | WLE |  | Negative | 50 | N |
| Nakazono (Nakazono et al., 2003) | 2003 | 1 | 32 | F |  | Parenchymal | XR, US, MRI | Y | Excision |  |  | 24 | N |
| Papantoniou (Papantoniou et al., 2005) | 2005 | 1 | 35 | F |  |  | XR | Y | Excision |  |  | 24 | Y |
| Plaza (Plaza & Yepes, 2012) | 2012 | 1 | 29 | F | Implant | Chest wall | XR, US, MRI | N | Incisional | Y |  | 14 | N/A |
| Privette (Privette et al., 2005) | 2005 | 1 | 75 | F |  | Chest wall | XR, US | N | Incisional | Y | Positive | 19 | N/A |
| Sachdeva (Sachdeva, 2011) | 2011 | 1 | 60 | F |  | Parenchymal | XR | Y | Excision |  |  | 12 | N |
| Samardzic (Samardzic et al., 2018) | 2018 | 1 | 65 | F |  |  | XR, US | N | Excision |  |  | 24 | N |
| Schwarz (Schwarz et al., 2006) | 2006 | 1 | 49 | F |  | Parenchymal | XR, US, MRI | N | WLE |  | Negative | 6 | N |
| Sheu (Sheu et al., 2022) | 2022 | 1 | 39 | M |  |  | XR, US | Y | Excision |  |  | 12 | N |
| Shim (Shim et al., 2014) | 2014 | 1 | 29 | F | Implant | Chest wall | US, MRI | Y | WLE |  |  | 8 | N |
| Steadman (Steadman & Crook, 2018) | 2018 | 1 | 25 | F |  | Chest wall | XR (neg), US, MRI | Y | WLE |  |  | 6 | N |
| Uttam (Uttam et al., 2021) | 2021 | 1 | 40 | M |  |  | PET | Y | WLE |  |  | 8 | N |
| Wongmaneerung (Wongmaneerung et al., 2016) | 2016 | 2 | 31 | F |  |  | XR (neg), US | N | Mastectomy |  |  | 84 | N |
|  |  |  | 28 | F |  | Parenchymal | US | N | Excision |  | Close | 12 | Y |
| Yamaguchi (Yamaguchi et al., 2002) | 2002 | 1 | 36 | F |  | Parenchymal | XR (neg), US | N | Excision |  | Positive | 36 | N |
| Yu (Yu et al., 2015) | 2015 | 1 | 70 | F |  | Chest wall | XR, US | N | WLE |  | Positive | 12 | N |

Table 1. Summary of the results of 46 patients with detailed case and follow-up information.

Abbreviations: XR – x-ray, US – ultrasound, WLE – wide local excision

References

Al-Khyatt, W., Goyal, A., & Mansel, R. E. (2010). Nipple-sparing skin-sparing mastectomy and vertical latissimus dorsi flap reconstruction for bilateral fibromatosis of the breast [Case Reports]. *Clinical Breast Cancer*, *10*(1), E1-2. <https://doi.org/https://dx.doi.org/10.3816/CBC.2010.n.012>

Al-Yusuf, R., Fakhro, A. R., & Alkhaznah, A. (2005). Breast fibromatosis [Review]. *Bahrain Medical Bulletin*, *27(4)*, 196-199. <https://access.ovid.com/custom/redirector/ciap.html?dest=https://acs.hcn.com.au/?acc=36422&url=https://ovidsp.ovid.com/ovidweb.cgi?T=JS&CSC=Y&NEWS=N&PAGE=fulltext&D=emed9&AN=41789558>

Benej, R., Meciarova, I., & Pohlodek, K. (2017). Desmoid-type fibromatosis of the breast: A report of 2 cases. *Oncology Letters*, *14(2)*, 1433-1438. <https://access.ovid.com/custom/redirector/ciap.html?dest=https://acs.hcn.com.au/?acc=36422&url=https://ovidsp.ovid.com/ovidweb.cgi?T=JS&CSC=Y&NEWS=N&PAGE=fulltext&D=emed18&AN=617154351>

Bouab, M., Harit, A., Boufettal, H., Mahdaoui, S., & Samouh, N. (2022). Desmoid fibromatosis of the breast occurring after breast reduction surgery mimicking a carcinoma: A rare case report. *Annals of Medicine and Surgery*, *77 (no pagination)*, Article 103526. <https://access.ovid.com/custom/redirector/ciap.html?dest=https://acs.hcn.com.au/?acc=36422&url=https://ovidsp.ovid.com/ovidweb.cgi?T=JS&CSC=Y&NEWS=N&PAGE=fulltext&D=emexb&AN=2017755959>

Brown, C. S., Jeffrey, B., Korentager, R., & Hughes, K. (2012). Desmoid tumors of the bilateral breasts in a patient without Gardner syndrome: a case report and review of literature [Case Reports

Review]. *Annals of Plastic Surgery*, *69*(2), 220-222. <https://doi.org/https://dx.doi.org/10.1097/SAP.0b013e31821e8faf>

Canan, A., & Wang, X. (2019). Recurrent desmoid tumor arising from latissimus dorsi flap: A case report [Case Reports]. *Clinical Imaging*, *53*, 191-194. <https://doi.org/https://dx.doi.org/10.1016/j.clinimag.2018.10.025>

Chummun, S., McLean, N. R., Abraham, S., & Youseff, M. (2010). Desmoid tumour of the breast [Case Reports]. *Journal of Plastic, Reconstructive & Aesthetic Surgery: JPRAS*, *63*(2), 339-345. <https://doi.org/https://dx.doi.org/10.1016/j.bjps.2008.09.024>

Collins, A. M., Granahan, A. M., Healy, D. G., Lawlor, C. A., & O'Neill, S. P. (2017). Giant desmoid tumour of the thorax following latissimus dorsi and implant breast reconstruction: case report and review of the literature [Case Reports

Review]. *Irish Medical Journal*, *110*(3), 534. <https://access.ovid.com/custom/redirector/ciap.html?dest=https://acs.hcn.com.au/?acc=36422&url=https://ovidsp.ovid.com/ovidweb.cgi?T=JS&CSC=Y&NEWS=N&PAGE=fulltext&D=med14&AN=28657247>

Foa, R., Rizzo, S., Petrella, F., De Maria, F., & Bellomi, M. (2014). Recurrent aggressive fibromatosis of the chest wall. *ecancermedicalscience*, *8 (no pagination)*, Article 464. <https://access.ovid.com/custom/redirector/ciap.html?dest=https://acs.hcn.com.au/?acc=36422&url=https://ovidsp.ovid.com/ovidweb.cgi?T=JS&CSC=Y&NEWS=N&PAGE=fulltext&D=emed15&AN=600047794>

Gergele, F., Guy, F., Collin, F., & Krause, D. (2012). A desmoid tumour associated with a breast prosthesis [Case Reports

Letter]. *Diagnostic and Interventional Imaging*, *93*(3), e200-203. <https://doi.org/https://dx.doi.org/10.1016/j.diii.2011.12.011>

Greenberg, D., McIntyre, H., Ramsaroop, R., Arthur, J., & Harman, J. (2002). Aggressive fibromatosis of the breast: a case report and literature review [Case Reports

Review]. *Breast Journal*, *8*(1), 55-57. <https://access.ovid.com/custom/redirector/ciap.html?dest=https://acs.hcn.com.au/?acc=36422&url=https://ovidsp.ovid.com/ovidweb.cgi?T=JS&CSC=Y&NEWS=N&PAGE=fulltext&D=med4&AN=11856165>

https://onlinelibrary.wiley.com/doi/abs/10.1046/j.1524-4741.2002.08013.x

Grimaldi, M. C., Trentin, C., Lo Gullo, R., & Cassano, E. (2018). Fibromatosis of the breast mimicking cancer: A case report. *Radiology Case Reports*, *13(1)*, 1-5. <https://access.ovid.com/custom/redirector/ciap.html?dest=https://acs.hcn.com.au/?acc=36422&url=https://ovidsp.ovid.com/ovidweb.cgi?T=JS&CSC=Y&NEWS=N&PAGE=fulltext&D=emed18&AN=619159110>

Hammood, Z. D., Salih, A. M., Kakamad, F. H., Abdullah, A. M., Ali, B. S., & Pshtiwan, L. R. A. (2021). Desmoid fibromatosis of the breast; a rare case report. *International Journal of Surgery Case Reports*, *87 (no pagination)*, Article 106363. <https://access.ovid.com/custom/redirector/ciap.html?dest=https://acs.hcn.com.au/?acc=36422&url=https://ovidsp.ovid.com/ovidweb.cgi?T=JS&CSC=Y&NEWS=N&PAGE=fulltext&D=emexa&AN=2014688445>

Hammoudeh, Z. S., & Darian, V. B. (2012). Desmoid tumor (fibromatosis) of the breast after augmentation with saline implants [Case Reports]. *Plastic & Reconstructive Surgery*, *129*(4), 753e-754e. <https://doi.org/https://dx.doi.org/10.1097/PRS.0b013e318245e918>

Henderson, P. W., Singh, S. P., & Spector, J. A. (2010). Chest wall spindle cell fibromatosis after breast augmentation [Case Reports]. *Plastic & Reconstructive Surgery*, *126*(2), 94e-95e. <https://doi.org/https://dx.doi.org/10.1097/PRS.0b013e3181de23ce>

Horevoets, J., Smet, B., Pattyn, P., van Dorpe, J., & Vuylsteke, P. (2013). Desmoid tumour of the breast: case report and review of the literature [Case Reports

Review]. *Acta Chirurgica Belgica*, *113*(4), 304-307. <https://access.ovid.com/custom/redirector/ciap.html?dest=https://acs.hcn.com.au/?acc=36422&url=https://ovidsp.ovid.com/ovidweb.cgi?T=JS&CSC=Y&NEWS=N&PAGE=fulltext&D=med10&AN=24224444>

Jung, H. K., Kim, E. K., Ko, K. H., & Kang, H. Y. (2010). Breast fibromatosis showing unusual sonographic features [Case Reports]. *Journal of Ultrasound in Medicine*, *29*(11), 1671-1674. <https://access.ovid.com/custom/redirector/ciap.html?dest=https://acs.hcn.com.au/?acc=36422&url=https://ovidsp.ovid.com/ovidweb.cgi?T=JS&CSC=Y&NEWS=N&PAGE=fulltext&D=med8&AN=20966482>

https://onlinelibrary.wiley.com/doi/abs/10.7863/jum.2010.29.11.1671?sid=nlm%3Apubmed

Karahan, O., Karanis, M. I. E., & Koksal, H. (2017). Synchronous breast and abdominal fibromatosis: A rare case. *Gazi Medical Journal*, *28(3)*, 210-211. <https://access.ovid.com/custom/redirector/ciap.html?dest=https://acs.hcn.com.au/?acc=36422&url=https://ovidsp.ovid.com/ovidweb.cgi?T=JS&CSC=Y&NEWS=N&PAGE=fulltext&D=emed18&AN=617033648>

Khanfir, K., Guinebretiere, J. M., Vanel, D., Barreau-Pouhaer, L., Bonvalot, S., & Le Cesne, A. (2003). Unusual problems in breast cancer and a rare lung cancer case. Case 2. Aggressive fibromatosis of the chest wall arising near a breast prosthesis [Case Reports]. *Journal of Clinical Oncology*, *21*(11), 2216-2218. <https://access.ovid.com/custom/redirector/ciap.html?dest=https://acs.hcn.com.au/?acc=36422&url=https://ovidsp.ovid.com/ovidweb.cgi?T=JS&CSC=Y&NEWS=N&PAGE=fulltext&D=med5&AN=12775750>

Kim, M. J., Wapnir, I. L., Ikeda, D. M., Chisholm, K. M., Do, Y., & Daniel, B. L. (2012). MRI enhancement correlates with high grade desmoid tumor of breast [Case Reports]. *Breast Journal*, *18*(4), 374-376. <https://doi.org/https://dx.doi.org/10.1111/j.1524-4741.2012.01255.x>

Lee, S. M., Lee, J. Y., Lee, B. H., Kim, S. Y., Joo, M., & Kim, J. I. (2015). Fibromatosis of the breast mimicking an abscess: case report of unusual sonographic features [Case Reports]. *Clinical Imaging*, *39*(4), 685-688. <https://doi.org/https://dx.doi.org/10.1016/j.clinimag.2015.03.002>

Lin, S., Cao, Y., Chen, L., Chen, M., Zhang, S., & Jia, X. (2021). Contrast-enhanced ultrasound of breast fibromatosis: a case report [Case Reports]. *Journal of International Medical Research*, *49*(5), 3000605211010619. <https://doi.org/https://dx.doi.org/10.1177/03000605211010619>

Matherne, T. H., Green, A., Jr., Tucker, J. A., & Dyess, D. L. (2004). Fibromatosis: the breast cancer imitator [Case Reports

Review]. *Southern Medical Journal*, *97*(11), 1100-1103. <https://access.ovid.com/custom/redirector/ciap.html?dest=https://acs.hcn.com.au/?acc=36422&url=https://ovidsp.ovid.com/ovidweb.cgi?T=JS&CSC=Y&NEWS=N&PAGE=fulltext&D=med5&AN=15586601>

Meshikhes, A. W., Butt, S., Al-Jaroof, A., & Al-Saeed, J. (2005). Fibromatosis of the male breast [Case Reports]. *Breast Journal*, *11*(4), 294. <https://access.ovid.com/custom/redirector/ciap.html?dest=https://acs.hcn.com.au/?acc=36422&url=https://ovidsp.ovid.com/ovidweb.cgi?T=JS&CSC=Y&NEWS=N&PAGE=fulltext&D=med6&AN=15982403>

https://onlinelibrary.wiley.com/doi/10.1111/j.1075-122x.2005.21558.x

Morales, R. D., Mendoza, A. G., Luces, C., Abreu, E. B., Romero, G., Perez, G., & Russo, L. (2018). Aggressive breast fibromatosis following augmentation mastoplasty: A series of case reports [Review]. *ecancermedicalscience*, *12 (no pagination)*, Article 833. <https://access.ovid.com/custom/redirector/ciap.html?dest=https://acs.hcn.com.au/?acc=36422&url=https://ovidsp.ovid.com/ovidweb.cgi?T=JS&CSC=Y&NEWS=N&PAGE=fulltext&D=emed19&AN=622174799>

Munhoz, A. M., Marques, A. D. A., Milanez, J. R., & Gemperli, R. (2017). Chest wall reconstruction following axillary breast augmentation and desmoid tumor resection using capsular flaps and a form-stable silicone implant: A case report, diagnosis and surgical technique. *International Journal of Surgery Case Reports*, *36*, 110-115. <https://access.ovid.com/custom/redirector/ciap.html?dest=https://acs.hcn.com.au/?acc=36422&url=https://ovidsp.ovid.com/ovidweb.cgi?T=JS&CSC=Y&NEWS=N&PAGE=fulltext&D=emed18&AN=616381461>

https://www.ncbi.nlm.nih.gov/pmc/articles/PMC5447517/pdf/main.pdf

Nakazono, T., Satoh, T., Hamamoto, T., & Kudo, S. (2003). Dynamic MRI of fibromatosis of the breast [Case Reports]. *AJR. American Journal of Roentgenology*, *181*(6), 1718-1719. <https://access.ovid.com/custom/redirector/ciap.html?dest=https://acs.hcn.com.au/?acc=36422&url=https://ovidsp.ovid.com/ovidweb.cgi?T=JS&CSC=Y&NEWS=N&PAGE=fulltext&D=med5&AN=14627606>

https://www.ajronline.org/doi/pdfplus/10.2214/ajr.181.6.1811718

Papantoniou, V., Koutsikos, J., Sotiropoulou, M., Feida, E., & Tsiouris, S. (2005). Recurrent bilateral mammary fibromatosis (desmoid tumor) imaged with technetium-99m pentavalent dimercaptosuccinic acid [99mTc-(V)DMSA] scintimammography [Case Reports]. *Gynecologic Oncology*, *97*(3), 964-969. <https://access.ovid.com/custom/redirector/ciap.html?dest=https://acs.hcn.com.au/?acc=36422&url=https://ovidsp.ovid.com/ovidweb.cgi?T=JS&CSC=Y&NEWS=N&PAGE=fulltext&D=med6&AN=15896828>

Plaza, M. J., & Yepes, M. (2012). Breast fibromatosis response to tamoxifen: dynamic MRI findings and review of the current treatment options [Case Reports]. *Journal of Radiology Case Reports*, *6*(3), 16-23. <https://doi.org/https://dx.doi.org/10.3941/jrcr.v6i3.897>

Privette, A., Fenton, S. J., Mone, M. C., Kennedy, A. M., & Nelson, E. W. (2005). Desmoid tumor: a case of mistaken identity [Case Reports]. *Breast Journal*, *11*(1), 60-64. <https://access.ovid.com/custom/redirector/ciap.html?dest=https://acs.hcn.com.au/?acc=36422&url=https://ovidsp.ovid.com/ovidweb.cgi?T=JS&CSC=Y&NEWS=N&PAGE=fulltext&D=med6&AN=15647081>

https://onlinelibrary.wiley.com/doi/10.1111/j.1075-122X.2005.21679.x

Sachdeva, S. (2011). Fibromatosis of breast mimicking sarcoidosis. *Indian Journal of Dermatology*, *56(3)*, 313-314. <https://access.ovid.com/custom/redirector/ciap.html?dest=https://acs.hcn.com.au/?acc=36422&url=https://ovidsp.ovid.com/ovidweb.cgi?T=JS&CSC=Y&NEWS=N&PAGE=fulltext&D=emed12&AN=362124927>

Samardzic, T., Lomo, J., & Skaane, P. (2018). Screening-detected desmoid tumor of the breast: findings at conventional imaging and digital breast tomosynthesis. *Acta Radiologica Open*, *7*(1). <https://access.ovid.com/custom/redirector/ciap.html?dest=https://acs.hcn.com.au/?acc=36422&url=https://ovidsp.ovid.com/ovidweb.cgi?T=JS&CSC=Y&NEWS=N&PAGE=fulltext&D=emed19&AN=620306624>

Schwarz, G. S., Drotman, M., Rosenblatt, R., Milner, L., Shamonki, J., & Osborne, M. P. (2006). Fibromatosis of the breast: case report and current concepts in the management of an uncommon lesion [Case Reports

Review]. *Breast Journal*, *12*(1), 66-71. <https://access.ovid.com/custom/redirector/ciap.html?dest=https://acs.hcn.com.au/?acc=36422&url=https://ovidsp.ovid.com/ovidweb.cgi?T=JS&CSC=Y&NEWS=N&PAGE=fulltext&D=med6&AN=16409590>

https://onlinelibrary.wiley.com/doi/10.1111/j.1075-122X.2006.00187.x

Sheu, T. C., Phung, S. C., Mammolito, D. M., & Guingrich, J. A. (2022). Fibromatosis of the breast in a male patient. *Radiology Case Reports*, *17(4)*, 1201-1204. <https://access.ovid.com/custom/redirector/ciap.html?dest=https://acs.hcn.com.au/?acc=36422&url=https://ovidsp.ovid.com/ovidweb.cgi?T=JS&CSC=Y&NEWS=N&PAGE=fulltext&D=emexb&AN=2016770998>

Shim, H. S., Kim, S. J., Kim, O. H., Jung, H. K., Kim, S. J., Kim, W., & Kim, W. W. (2014). Fibromatosis associated with silicone breast implant: ultrasonography and MR imaging findings [Case Reports]. *Breast Journal*, *20*(6), 645-649. <https://doi.org/https://dx.doi.org/10.1111/tbj.12340>

Steadman, L., & Crook, S. (2018). Fibromatosis arising from the pectoralis major muscle mimicking breast cancer. *Radiology Case Reports*, *13(6)*, 1174-1178. <https://access.ovid.com/custom/redirector/ciap.html?dest=https://acs.hcn.com.au/?acc=36422&url=https://ovidsp.ovid.com/ovidweb.cgi?T=JS&CSC=Y&NEWS=N&PAGE=fulltext&D=emed19&AN=2001101856>

Uttam, P., Gupta, R. K., & Hussain, N. (2021). Male breast fibromatosis [Case Reports

Letter]. *Indian Journal of Pathology & Microbiology*, *64*(2), 430-432. <https://doi.org/https://dx.doi.org/10.4103/IJPM.IJPM_649_19>

Wongmaneerung, P., Somwangprasert, A., Watcharachan, K., & Ditsatham, C. (2016). Bilateral desmoid tumor of the breast: Case series and literature review [Review]. *International Medical Case Reports Journal*, *9*, 247-251. <https://access.ovid.com/custom/redirector/ciap.html?dest=https://acs.hcn.com.au/?acc=36422&url=https://ovidsp.ovid.com/ovidweb.cgi?T=JS&CSC=Y&NEWS=N&PAGE=fulltext&D=emed17&AN=611766167>

https://www.dovepress.com/getfile.php?fileID=32000

Yamaguchi, H., Sakakibara, T., Hino, M., Ryu, M., Senuma, K., Nakai, K., Tomiki, Y., Sakamoto, K., Kamano, T., Tsurumaru, M., & Matsumoto, T. (2002). A case of fibromatosis of the breast [Case Reports

Review]. *Breast Cancer*, *9*(2), 175-178. <https://access.ovid.com/custom/redirector/ciap.html?dest=https://acs.hcn.com.au/?acc=36422&url=https://ovidsp.ovid.com/ovidweb.cgi?T=JS&CSC=Y&NEWS=N&PAGE=fulltext&D=med4&AN=12016399>

Yu, W. L., Wu, C., & Chau, H. H. L. (2015). Mimicker of breast cancer: Mammary fibromatosis. *Hong Kong Journal of Radiology*, *18(2)*, 156-159. <https://access.ovid.com/custom/redirector/ciap.html?dest=https://acs.hcn.com.au/?acc=36422&url=https://ovidsp.ovid.com/ovidweb.cgi?T=JS&CSC=Y&NEWS=N&PAGE=fulltext&D=emed16&AN=605518079>
